# Quantitative assessment of left ventricular myocardial involvement in patients with connective tissue disease: a 3.0T contrast-enhanced cardiovascular magnetic resonance study

**DOI:** 10.1007/s10554-022-02539-6

**Published:** 2022-03-13

**Authors:** Jin Wang, Yue Gao, Zhi-Gang Yang, Ying-Kun Guo, Li Jiang, Rui Shi, Hua-Yan Xu, Shan Huang, Yuan Li

**Affiliations:** 1 Department of Radiology, West China Hospital, Sichuan University, 37# Guo Xue Xiang, Chengdu, 610041 Sichuan China; 2 Department of Radiology, Key Laboratory of Obstetric & Gynecologic and Pediatric Diseases and Birth Defects of Ministry of Education, West China Second University Hospital, Sichuan University, 20# Section 3, Renmin South Road, Chengdu, 610041 Sichuan China

**Keywords:** Connective tissue disease, Left ventricular, Cardiovascular magnetic resonance, Strain, Perfusion, Late gadolinium enhancement

## Abstract

The aim of this study was to evaluate left ventricular (LV) myocardial involvement in connective tissue disease (CTD) patients using multiparemetric imaging derived from cardiovascular magnetic resonance (CMR). CMR was performed on 146 CTD patients (comprising of 74 with idiopathic inflammatory myopathy (IIM) and 72 with non-IIM) and 72 healthy controls and included measures of LV global strains [including peak strain (PS), peak systolic (PSSR) and diastolic strain rate (PDSR)], myocardial perfusion [including upslope, max signal intensity (MaxSI), and time to maximum signal intensity (TTM)], and late gadolinium enhancement (LGE) parameters. Univariable and multivariable linear regression analyses were performed to determine the association between LV deformation and microvascular perfusion, as well as LGE. Our results indicated that CTD patients had decreased global longitudinal PS (GLPS), PSSR, PDSR, and myocardial perfusion (all p < 0.017) compared with normal controls. Non-IIM patients exhibited lower LV global strain and longer TTM than IIM patients. The presence of LGE was independently associated with global radial PS (GRPS: β = − 0.165, p = 0.011) and global circumferential PS (GCPS: β =  − 0.122, p = 0.022). TTM was independently correlated with GLPS (β = − 0.156, p = 0.027). GLPS was the best indicator for differentiating CTD patients from normal controls (area under curve of 0.78). This study indicated that CTD patients showed impaired LV global myocardial deformation and microvascular perfusion, and presence of LGE. Cardiac involvement might be more severe in non-IIM patients than in IIM patients. Impaired microvascular perfusion and the presence of LGE were independently associated with LV global deformation.

**Supplementary Information:**

The online version contains supplementary material available at 10.1007/s10554-022-02539-6.

## Introduction

Connective tissue disease (CTD) encompasses a group of systematic inflammatory diseases, including idiopathic inflammatory myopathy (IIM), systemic lupus erythematosus (SLE), rheumatoid arthritis (RA), and systemic sclerosis (SSc), all of which may share common pathogenic mechanisms and multiorgan involvement including the heart [1–4]. Cardiac involvement in patients with CTD represents one of the leading causes of death [4, 5]. This population may manifest with varying degrees of clinical presentations, most of which are subclinical and progress gradually [5, 6]. Once cardiac involvement develops into clinical heart failure, patients will carry an ominous prognosis [4, 7]. Thus, early identification of cardiac abnormalities is key to increasing the possibility of early treatment for improvement of long-term outcomes and survival [8].

Cardiovascular magnetic resonance (CMR) imaging is the most reliable non-invasive technique, which is considered the reference standard for the quantitative assessment of cardiac geometry and function, myocardial perfusion, as well as tissue characteristics with high spatial and temporal resolution [9–11]. Multiparametric imaging derived from CMR has been increasingly used to evaluate a variety of cardiac diseases including ischemic cardiomyopathy and non-ischemic cardiomyopathy resulting from diabetes mellitus, amyloidosis, and hypertension [12–14]. However, to the best of our knowledge, few studies have simultaneously evaluated myocardial strain, microcirculation perfusion and myocardial fibrosis in patients with CTD [15, 16]. Therefore, the present study aimed to quantitatively assess the multiple parameters derived from CMR including LV global myocardial deformation, microvascular perfusion, and late gadolinium enhancement (LGE) in CTD patients and investigated the association between LV deformation and myocardial perfusion, as well as LGE.

## Materials and methods

### Study population

Between January 2015 and July 2021, 183 patients with CTD at our hospital were retrospectively enrolled in this study. Those patients underwent 3.0T CMR examination in order to assess, quantify and early detect cardiac involvement based on the current recommendations of the International Consensus Group on CMR in Rheumatology [4]. Diagnosis of CTD was based on the criteria of the American College of Rheumatology or the European League Against Rheumatism, respectively [3]. The detailed diagnostic criteria for CTD [17–23] were shown in the Supplementary Information*. *Exclusion criteria included congenital heart disease, heart valve disease, cardiomyopathy, coronary artery disease, and severe liver, lung, and kidney dysfunction. Finally, a total of 146 patients with CTD (mean age, 45.12 ± 13.52 years; 114 female) were included in this study, comprising 74 patients with IIM and 72 patients with non-IIM (39 with SLE, 7 with RA, 3 with SSc, 8 with mixed connective tissue disease, 9 with Sjogren's syndrome, and 6 with undifferentiated connective tissue disease). Seventy-two age- and gender-matched healthy individuals (mean age, 47.06 ± 11.93 years; 48 female) were selected to serve as the normal control group with no history of cardiovascular or systematic disease that underwent 3.0T CMR during the same period. The clinical marker N-terminal pro-brain natriuretic peptide (NT-proBNP) was examined in all participants. This study protocol was approved by our hospital of Biomedical Research Ethics Committee (No. 2019-756) and conducted in accordance with the ethical guidelines of the Declaration of Helsinki, and all participants provided informed consent.

### CMR protocol

In a supine position, all participants were examined using a 3.0T whole-body scanner (Trio Tim; Siemens Medical Solutions, Erlangen, Germany). Continuous data acquisition was performed using the manufacturer’s standard ECG-triggering device which monitored dynamic changes in each individual’s ECG findings during the breath-holding period. From the base to the apex, 8–12 continuous CMR cine images of the long-axis (two- and four-chamber) and short-axis views were acquired using a balanced steady-state free precession (bSSFP) sequence (TR/TE 3.4/1.22 ms, field of view 340 × 284.42 mm, flip angle 38°, slice thickness 8 mm, and matrix size 256 × 166). Subsequently, a dose of 0.2 mL/kg gadobenate dimeglumine (MultiHance 0.5 mmol/mL; Bracco, Milan, Italy) was intravenously injected using an automated injector (Stellant, MEDRAD, Indianola, PA, USA) at a flow rate of 2.5−3.0 mL/s, followed by a 20 mL saline flush immediately injected at a rate of 3.0 mL/s. The first-pass perfusion images were obtained in one slice of the four-chamber view and in three standard short-axis slices (the basal, middle, and apical) performed with an inversion recovery prepared echo-planar imaging sequence (TR/TE 154.38/1.07 ms, flip angle 10°, slice thickness 8 mm, field of view 340 mm × 255 mm, and matrix size 256 × 192). LGE images were achieved at 10−15 min after contrast administration using segmented−turbo−FLASH−phase-sensitive inversion recovery (PSIR) sequences (TR/TE 583 ms/1.4 ms, flip angle 40°, slice thickness 8 mm, field of view 360 × 270 mm, and matrix size 256 × 148).

### CMR image analysis

CMR images were analyzed offline using commercial software (cvi42, Circle Cardiovascular Imaging Inc., Calgary, AB, Canada) by two experienced radiologists, each of whom had more than 4 years of CMR experience.

The endocardial and epicardial borders of the LV myocardium were manually outlined in the serial short-axis cine images at the end-diastolic and end-systolic periods using the aforementioned software. Then, cardiac geometry and function parameters including LV ejection fraction (LVEF), LV end-diastolic volume (LVEDV), LV end-systolic volume (LVESV), LV stroke volume (LVSV), LV mass (LVM), and LV remodeling index (calculated as LVM/LVEDV) were calculated according to the current guideline [24]. The papillary muscles and moderator bands were included in the LV cavity, but they were excluded from the LV mass.

**Fig. 1:**
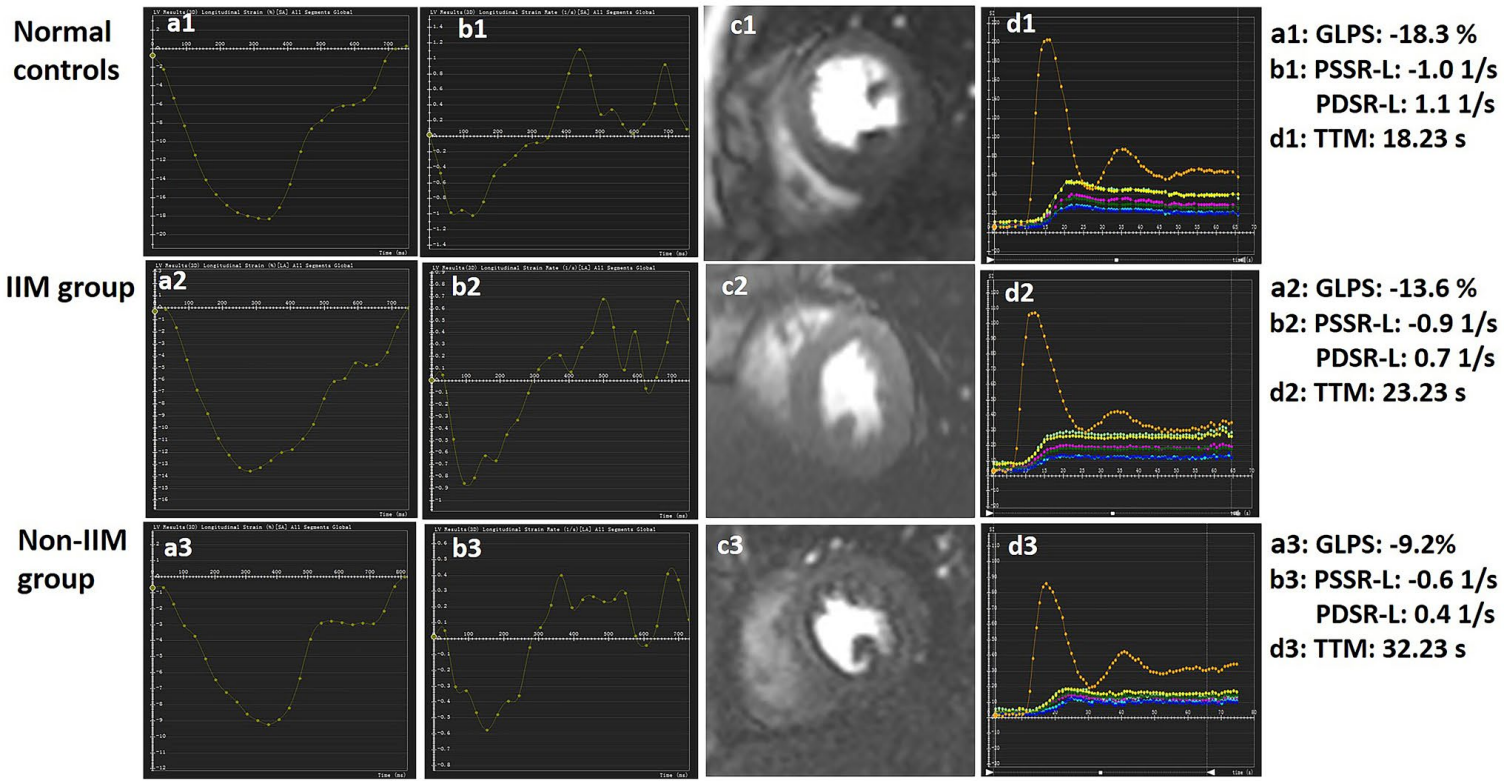
Cardiovascular magnetic resonance (CMR) images analyses among normal controls, IIM and non-IIM groups. Representative CMR-derived parametric images including the global peak strain curves (a1, a2, and a3) and global peak strain rate curves (b1, b2, and b3) in longitudinal direction, left ventricular first-pass perfusion images in mid-ventricular slice (c1, c2, and c3), and first-pass perfusion signal intensity-time curves (d1, d2, and d3) in a normal control, IIM patient and non-IIM patient. *GLPS* global longitudinal peak strain, *PSSR-L* The longitudinal peak systolic strain rate, *PDSR-L* the global longitudinal diastolic strain rate, *TTM* time to maximum signal intensity, *IIM* idiopathic inflammatory myopathy

A set of long-axis four-chamber and short-axis slices were loaded into the tissue tracking module to evaluate LV myocardial strain. In all series, the endocardial and epicardial borders of LV were delineated manually in each slice at the end-diastolic period (reference phase) with the papillary muscles and moderator bands excluded. Subsequently, three-dimensional (3D) tissue tracking parameters including the global radial, circumferential, and longitudinal peak strain (PS), peak systolic strain rate (PSSR), and peak diastolic strain rate (PDSR) were obtained automatically (e.g. Figure 1: a1, a2, and a3; b1, b2, and b3).

For the analysis of first-pass myocardial perfusion, endocardium, epicardium, and blood pool contours of all three slices of first-pass perfusion images (the apical, middle, and basal) were delineated manually with exclusion of papillary muscles and moderator bands. Each myocardial segment based on the 16-segment model (Bull’s eye plot) and the blood pool signal intensity-time curves were generated. Consequently, each myocardial segmental perfusion parameters including the upslope, maximum signal intensity (MaxSI), and time to maximum signal intensity (TTM) were obtained automatically from the myocardial signal intensity-time curves (Fig. 1: d1, d2, and d3). The global first-pass myocardial perfusion parameters were calculated by averaging the segmental values of the 16 myocardial segments. The presence or absence of LGE was evaluated by the two experienced CMR radiologists who had been blinded to patient information. A threshold, defined as five standard deviations (SDs) above the signal of the remote normal myocardial region, was used to measure the extent of LGE [25].

### Reproducibility of LV global myocardial strain and perfusion

The intra- and inter-observer variabilities for LV global myocardial strain and perfusion parameters were obtained randomly in 40 individuals including 30 CTD patients and 10 healthy controls. Intra-observer variability was determined by comparing the strain and perfusion indices by the same observer with a 1-month interval. Inter-observer variability was calculated by comparing the independent measurements of two double-blinded and experienced observers with more than four years of CMR experience.

### Statistical analysis

Statistical analyses were performed using IBM SPSS Statistics for Windows (version 24.0; IBM Corporation, Armonk, NY, USA). The Shapiro–Wilk test was used to evaluate data for normality and Levene's test for homogeneity of variance. Normally distributed data were expressed as the mean ± standard deviation (SD), and non-parametric variables were expressed as the median (interquartile range, 25%–75%). The CMR-derived parameters between CTD patients and healthy controls were compared using a Student’s *t*-test or Mann–Whitney U test. Parameters among controls, IIM, and non-IIM groups were compared by one-way analysis of variance (One-way ANOVA) followed by Bonferroni’s post hoc-test or the Kruskal–Wallis rank test, appropriately. Univariable linear regression analyses were performed to show the relationship between LV global PS and perfusion, LGE, and statistically significant CMR indices, as well as clinical variables. Variables with *p* values of less than 0.1 in the univariable analyses were included in stepwise multivariable linear regression models. Receiver operating characteristic (ROC) analysis was used to obtain the best discriminating parameters in global longitudinal strain and TTM to differentiate CTD patients from normal controls. Intra- and inter-observer variabilities for reproducibility were assessed using the intra-class correlation coefficient (ICC). A two-tailed p value of < 0.05 was considered statistically significant.

## Results

### Baseline characteristics and LV geometry and function

The baseline characteristics and LV geometry and function of the study participants are shown in Table 1. Of the 146 CTD patients, 114 (78.08%) patients were female and the median disease duration was 0.63 years. Age, BMI, BSA, heart rate, SBP and DBP were not significantly different between CTD patients and normal controls (all *p* > 0.05), except for a higher NT-proBNP levels in both the IIM [125 (48, 234) vs. 51(29, 60) pg/ml] and non-IIM [711 (224, 3269) vs. 51(29, 60) pg/ml] groups than in the control group (all p < 0.05).

**Table 1:** Baseline characteristics of the study cohort

	Normal controls	CTD	CTD	
	(n = 72)	(n = 146)	IIM (n = 74)	non-IIM (n = 72)
Age (years)	47.06 ± 11.93	45.12 ± 13.52	47.34 ± 13.28	42.84 ± 13.49*§
Female (n) %	48 (66.67%)	114 (78.08%)	50 (67.57%)	64 (88.89%)*
BMI (Kg/m^2^)	21.02 ± 2.86	21.75 ± 2.92	21.79 ± 2.67	21.71 ± 3.16
BSA (m^2^)	1.58 ± 0.15	1.56 ± 0.16	1.57 ± 0.16	1.54 ± 0.16
Systolic blood pressure (mmHg)	115.31 ± 5.86	119.21 ± 18.46	122.11 ± 18.16*	116.24 ± 18.41
Diastolic blood pressure (mmHg)	75.17 ± 3.99	78.16 ± 13.02	78.20 ± 11.88	78.13 ± 14.18
Heart rate (beats/min)	73.28 ± 4.03	74.52 ± 10.39	77.23 ± 11.47*	71.74 ± 8.33§
Disease duration (years)	–	0.63 (0.27, 3.62)	0.49 (0.21, 1.50)	2 (0.38, 10.10) §
NT-proBNP (pg/mL)	51 (29,60)	226 (111, 868)†	125 (48, 234) *	711 (224, 3269)*§
CMR findings				
LVEF (%)	62.55 ± 7.15	58.57 ± 13.23†	62.57 ± 7.08	54.47 ± 16.49*§
LVEDV (mL)	120.93 ± 21.73	116.31 ± 32.92	117.94 ± 29.27	114.52 ± 36.69
LVESV (mL)	45.07 ± 11.81	46.95 ± 21.98	44.36 ± 14.69	49.87 ± 27.83*§
LVSV (mL)	75.48 ± 16.51	66.55 ± 18.90†	70.66 ± 15.36	62.50 ± 21.18*§
LVM (g)	67.63 ± 19.30	85.19 ± 23.44†	87.15 ± 23.61*	86.05 ± 26.76*
LV remodeling index (g/mL)	0.56 ± 0.13	0.76 ± 0.19†	0.77 ± 0.21*	0.76 ± 0.19*

All values are presented as mean ± SD or n (%) or interquartile range

*CTD* connective tissue disease, *IIM* idiopathic inflammatory myopathy, *BSA* body surface area, *BMI* body mass index, *NT-proBNP* N-terminal pro-brain natriuretic peptide, *CMR* cardiovascular magnetic resonance, *LV* left ventricular, *EF* ejection fraction, *EDV* end-diastolic volume, *ESV* end-systolic volume, *SV* stroke-volume

†p < 0.05 CTD patients versus normal controls, *p < 0.05 versus normal controls, §p < 0.05 versus IIM patients

Compared to the healthy control group, CTD patients had decreased LVEF and LVSV, meanwhile increased LVM and LV remodeling index. The non-IIM group had significantly lower LVEF and LVSV, and higher LVESV than normal subjects and IIM patients (all p < 0.05). In both of the IIM and non-IIM groups, the LVM and LV remodeling index were increased compared to healthy controls (both p < 0.05); however, there was no significant difference in them between IIM and non-IIM groups (both *p* > 0.05) (Table 1).

### Comparison of CMR-derived LV global strain, microvascular perfusion, and LGE between CTD patients and normal controls

CMR findings for the observed groups are summarized in Table 2, and case examples are shown in Fig. 1. In comparison with normal controls, CTD patients demonstrated disturbances in LV myocardial strain, which mainly involved the global PS in the three directions, global longitudinal PSSR (PSSR-L), and PDSR (PDSR-L) (all p < 0.017); besides, impaired LV myocardial perfusion was also observed in CTD patients manifesting as reduced upslope (2.37 ± 0.65 vs. 2.73 ± 0.66, p < 0.017) and MaxSI (22.48 ± 5.89 vs. 26.17 ± 4.12, p < 0.017), and an increased TTM (28.42 ± 5.56 vs. 24.43 ± 4.12, p < 0.017).

**Table 2:** Comparison of cardiac parameters between CTD patients and normal controls

	Normal controls	CTD	CTD	
	(n = 72)	(n = 146)	IIM (n = 74)	Non-IIM (n = 72)
PS (%)				
GRPS	35.09 ± 7.57	30.82 ± 12.09†	36.01 ± 9.53	25.48 ± 12.16*§
GCPS	− 20.68 ± 2.51	− 19.62 ± 4.89†	− 21.42 ± 3.48	− 17.77 ± 5.44*§
GLPS	− 14.33 ± 2.30	− 10.92 ± 3.87†	− 12.45 ± 3.80*	− 9.35 ± 3.29*§
PSSR (1/s)				
Radial	2.09 ± 0.80	1.90 ± 0.84	2.28 ± 0.74	1.52 ± 0.76*§
Circumferential	− 1.04 ± 0.21	− 1.06 ± 0.37	− 1.16 ± 0.43	− 0.97 ± 0.28§
Longitudinal	− 0.95 ± 0.26	− 0.76 ± 0.33†	− 0.83 ± 0.21*	− 0.68 ± 0.41*§
PDSR (1/s)				
Radial	− 2.47 ± 0.69	− 2.23 ± 1.01†	− 2.64 ± 0.96	− 1.81 ± 0.89*§
Circumferential	1.29 ± 0.23	1.27 ± 0.38	1.42 ± 0.34	1.12 ± 0.36*§
Longitudinal	1.04 ± 0.20	0.83 ± 0.31†	0.93 ± 0.29*	0.72 ± 0.31*§
Myocardial perfusion				
Upslope	2.73 ± 0.66	2.37 ± 0.65†	2.43 ± 0.65*	2.32 ± 0.66*
MaxSI	26.17 ± 4.12	22.48 ± 5.89†	23.66 ± 6.09*	21.25 ± 5.44*
TTM (s)	24.43 ± 4.12	28.42 ± 5.56†	27.01 ± 5.44*	29.47 ± 5.60*§
LGE				
LGE, n (%)	–	27 (18.5%)	10 (13.5%)	17 (23.6%)*
LGE rel (%)	–	1.1 (0.07, 3.31)%	0.35 (0.02, 1.59)%	2.59 (0.64, 7.35)%*

*Notes* All values are presented as mean ± SD or n (%) or interquartile range

*CTD* connective tissue disease, *IIM* idiopathic inflammatory myopathy, *PS* peak strain, *GRPS* global radial peak strain, *GCPS* global circumferential peak strain, *GLPS* global longitudinal peak strain, *PSSR* peak systolic strain rate, *PDSR* peak diastolic strain rate, *MaxSI* max signal intensity, *TTM* time to maximum signal intensity, *LGE* late gadolinium enhancement

^†^p < 0.05 CTD patients versus normal group, *p < 0.017 versus normal group, §p < 0.017 versus IIM patients

Non-IIM patients had lower global strain parameters including PS, PSSR, and PDSR in the three directions and longer TTM (29.47 ± 5.60 vs. 27.01 ± 5.44 s, p < 0.017) than IIM patients. There were no significant differences between IIM and non-IIM patients in terms of upslope and MaxSI (both *p* > 0.05). Twenty-seven patients (18.5%) were LGE-positive with a median extent of 1.1 (0.07, 3.31) % by the threshold of 5SD. Non-IIM patients exhibited a higher presence [17(23.6%) vs. 10 (13.5%), p < 0.017] and higher extent of LGE [2.59 (0.64, 7.35) % vs. 0.35 (0.02, 1.59) %, p < 0.017) than IIM patients.

### Correlations between LV global myocardial PS and clinical factors, as well as imaging variables in CTD

The associations between LV global PS and different clinical factors, as well as CMR parameters in CTD are demonstrated in Table 3. Increasing NT-proBNP was significantly associated with worsening GRPS (r = − 0.465, p < 0.05), GCPS (r = − 0.481, p < 0.05), and GLPS (r = − 0.539, p < 0.05). LV remodeling index was weakly correlated with GLPS (r = − 0.179, p < 0.05). The extent of LGE was significantly associated with GRPS (r = − 0.252, p < 0.05) and GCPS (r = − 0.231, p < 0.05).

**Table 3:** Univariable and multivariable linear regression analyses in patients with CTD

	GRPS (%)		GCPS (%)		GLPS (%)	
	Univariable r	Multivariable β	Univariable r	Multivariable β	Univariable r	Multivariable β
		R^2^ = 0.470		R^2^ = 0.664		R^2^ = 0.349
Disease duration	− 0.134	–	− 0.115	–	− 0.183*	–
NT-proBNP	− 0.465*	–	− 0.481*	–	− 0.539*	− 0.225^§^
EDV	− 0.312*	− 0.698^§^	− 0.305*	− 0.688^§^	− 0.175*	–
SV	0.227*	0.478^§^	0.235*	0.570^§^	0.251*	0.290^§^
LVM	− 0.352*	–	− 0.403*	− 0.199^§^	− 0.39*	− 0.395^§^
LV remodeling index	0.026	–	− 0.026	–	− 0.179*	–
LGE presence	− 0.318*	− 0.165^§^	− 0.337*	− 0.122^§^	− 0.257*	–
LGE extent	− 0.252*	–	− 0.231*	–	− 0.145*	–
TTM	− 0.169*	–	− 0.193*	–	− 0.283*	− 0.156^§^

Factors with p < 0.1 in the univariable analyses were included in the stepwise multivariable analyses

β Standardized coefficient

*CTD* connective tissue disease, *GRPS* global radial peak strain, *GCPS* global circumferential peak strain, *GLPS* global longitudinal peak strain, *NT-proBNP* N-terminal pro-brain natriuretic peptide, *EDV* end-diastolic volume; *ESV* end-systolic volume, *SV* stroke-volume, *LV* left ventricular, *LVM* left ventricular mass, *LGE* late gadolinium enhancement, *TTM* time to maximum signal intensity

*p < 0.1, §p < 0.05

Multivariable linear regression analyses revealed that considering the covariates of disease duration, NT-proBNP levels, and CMR function parameters, TTM was independently associated with the GLPS (β = − 0.156, p = 0.027, model R^2^ = 0.349). In addition, the presence of LGE was independently correlated with the GRPS (β = − 0.165, p = 0.011, model R^2^ = 0.470) and GCPS (β = − 0.122, p = 0.022, model R^2^ = 0.664) (Table 3).

### ROC curve analysis of the LV global longitudinal strain and TTM for discriminating CTD patients from normal controls

**Fig. 2:**
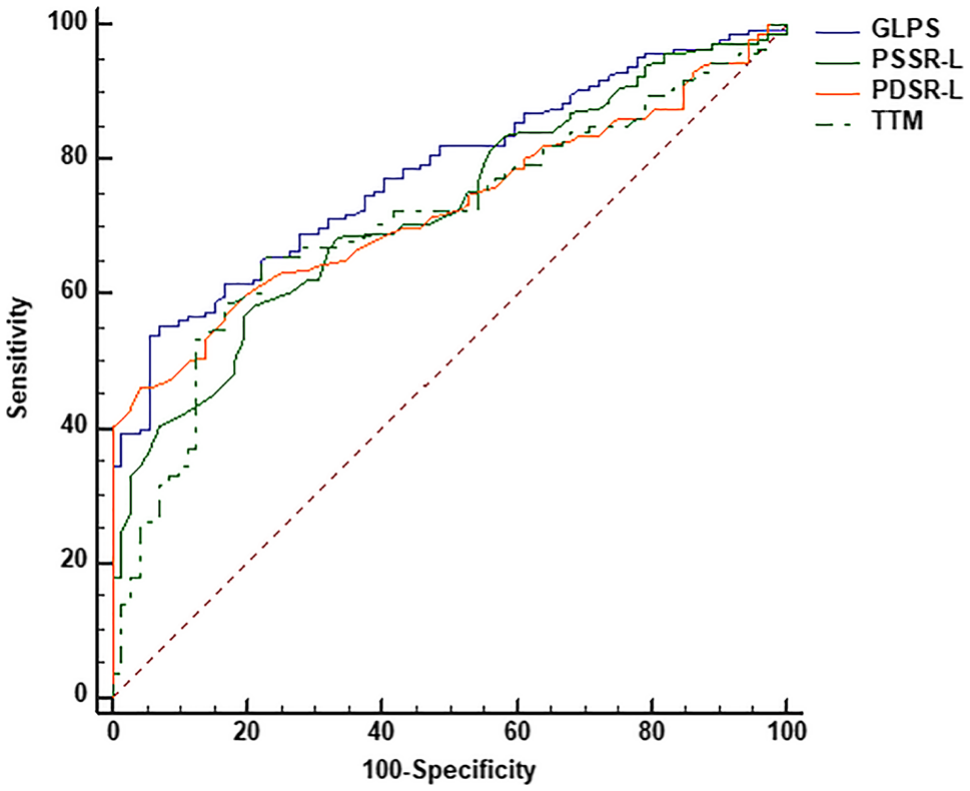
The Receiver operating characteristic (ROC) analysis between CTD patients and normal controls. Use of cut-off values of the GLPS (blue), PSSR-L (green), PDSR-L (orange), and TTM (the green dotted line) could discriminate CTD patients from that of normal controls. *GLPS* global longitudinal peak strain, *PSSR-L* the longitudinal peak systolic strain rate, *PDSR-L* the global longitudinal diastolic strain rate, *TTM* time to maximum signal intensity, *CTD* connective tissue disease

The LV GLPS, PSSR-L, PDSR-L, and TTM had moderate efficiencies in discriminating CTD patients from healthy controls [GLPS: area under the curve (AUC) 0.780, sensitivity 54.11%, specificity 94.44%, cut-off value − 11.45%; PSSR-L: AUC 0.725, sensitivity 56.85%, specificity 80.56%, cut-off value − 0.78 1/s; PDSR-L: AUC 0.728, sensitivity 45.89%, specificity 95.83%, cut-off value 0.77 1/s; TTM: AUC 0.709, sensitivity 65.75%, specificity 77.78%, cut-off value 26.15 s; all p < 0.001] (Fig. 2).

### Inter-observer and intra-observer variability

As shown in Table 4, there were fantastic inter- and intra- observer agreements in the measurement of LV global myocardial PS (ICC = 0.905−0.960 and 0.900−0.969, respectively), PSSR (ICC = 0.893–0.909 and 0.901–0.939, respectively), and PDSR (ICC = 0.895–0.944 and 0.908–0.951, respectively), as well as first-pass myocardial perfusion (ICC = 0.942–0.975 and 0.965–0.981, respectively).

**Table 4:** Inter-and intra-observer variabilities of LV myocardial strain and perfusion parameters

	Inter-observer (n = 40)		Intra-observer (n = 40)	
	ICC	95% CI	ICC	95% CI
PS (%)				
GRPS	0.932	0.859–0.966	0.969	0.943–0.984
GCPS	0.960	0.926–0.979	0.951	0.900–0.975
GLPS	0.905	0.829–0.949	0.900	0.820–0.946
PSSR (1/s)				
Radial	0.893	0.803–0.943	0.925	0.863–0.959
Circumferential	0.909	0.836–0.951	0.939	0.888–0.967
Longitudinal	0.896	0.812–0.944	0.901	0.822–0.947
PDSR (1/s)				
Radial	0.944	0.882–0.972	0.951	0.909–0.974
Circumferential	0.902	0.823–0.947	0.932	0.875–0.963
Longitudinal	0.895	0.804–0.944	0.908	0.834–0.950
Myocardial perfusion				
Upslope	0.972	0.946–0.986	0.965	0.881–0.985
MaxSI	0.942	0.893–0.969	0.971	0.946–0.984
TTM (s)	0.975	0.953–0.987	0.981	0.965–0.990

*ICC* intraclass correlation coefficient, *CI* confidence interval, *PS* peak strain, *GRPS* global radial peak strain, *GCPS* global circumferential peak strain, *GLPS* global longitudinal peak strain, *PSSR* peak systolic strain rate, *PDSR* peak diastolic strain rate, *MaxSI* max signal intensity, *TTM* time to maximum signal intensity

## Discussion

A wide range of CTD shares potential cardiac involvement, including IIM and non-IIM, such as SLE, RA, and SSc [3, 9, 15]. The underlying mechanisms of myocardial involvement can either result from direct inflammatory myocardial injury or be mediated via vasculitic coronary involvement, endothelial dysfunction, or microvascular disease [26, 27]. In the present study, we combined multiparametric CMR imaging to quantitatively evaluate cardiac abnormalities in a group of CTD patients. The following principal findings were obtained: (1) CTD patients (both in the IIM and non-IIM groups) had impaired LV global strain and microvascular perfusion, as well as presented with LGE. (2) LV deformation and microcirculation perfusion impaired more severe, as well as higher presence and extent of LGE in non-IIM patients than those in IIM patients. (3) LV global deformation showed correlations with the presence of LGE and TTM. 4) ROC analysis indicated the GLPS was the best indicator in differentiating in CTD patients from normal controls.

Accumulating data suggest that myocardial deformation measures (e.g. peak strain and strain rate) are reliable indices of ventricular systolic and diastolic function [28–30]. Our CTD patients exhibited deteriorated LV global strain that mainly involved the PS in the three directions, the longitudinal PDSR and PSSR, and the radial PDSR compared to normal controls, which suggested the impairment of both LV systolic and diastolic function in CTD. Our study also provided evidence that CTD patients had decreased LV myocardial perfusion (manifesting as the reduction of upslope and MaxSI, as well as increase of TTM) and presented with LGE, which were similar to previous reports in other CTD groups [3, 15, 16, 31]. On the one hand, chronic inflammation and immune deregulation in CTD contribute to impaired endothelial tissue and disruption in the myocardial interstitial matrix as a result of microvascular ischemia [4, 32, 33]. On the other hand, prolonged inflammation leads to oxidative stress and a cytokine-induced increase in fibroblast activity causing the accumulation of myocardial collagen degradation products and interstitial fibrosis [27, 33, 34], which could explain the results described above.

Studies in different disease settings, including IIM and non-IIM (such as SLE, RA, and SSc), revealed early myocardial perfusion defects coexisting with normal coronary arteries, significantly lower global strain, and LGE-positive states evaluated by CMR [16, 31, 35–37]. Being similar to such studies, the current research revealed that, in contrast to normal controls, both subgroups of IIM and non-IIM patients demonstrated impaired LV global deformation (involving the GLPS, PSSR-L, and PDSR-L) and lower myocardial perfusion (manifesting as reduced global upslope, MaxSI, and increased TTM), as well as presence of LGE. Our data showed more severe impairments of LV deformation and microvascular perfusion, as well as higher presence and extent of LGE in non-IIM patients than those in IIM patients. We speculated that the precise mechanisms of myocardial impairment in non-IIM might be different from that in IIM. In addition, disease duration in our non-IIM patients was longer than that in IIM patients, which might result in more severe myocardial injury in non-IIM patients than that in IIM patients. Further researches are still needed to understand the precise reasons for the severity of cardiac involvement between IIM and non-IIM.

CMR perfusion parameters including upslope, MaxSI, and TTM which derived from the CMR signal intensity-time curve and reflected myocardial perfusion reserve, are correlated with coronary microvascular function [28, 38]. Besides, CMR-derived LGE has been validated as an imaging marker to evaluate myocardial fibrosis [39]. Given the longitudinal strain is predominantly influenced by the longitudinally oriented subendocardial myocardial fibers, which are most susceptible to ischemia [40, 41], and deformation parameters have been reported to correlate with CMR-derived LGE [42] which usually occurs in the mid-myocardial and epicardial layer, as reported by a series of CTD studies [3, 16, 43]. We assumed that the impaired coronary microcirculation function and myocardial fibrosis might conduce to the myocardial dysfunction presenting as impaired deformation. Our results were in agreement with previous data, which showed that TTM was independently associated with GLPS. Besides, our data also demonstrated that the presence of LGE was independently correlated with the GRPS and GCPS. These findings could support the above hypothesis and indicate a potential mechanistic link between coronary microvascular dysfunction, myocardial fibrosis, and abnormal LV deformation. Additionally, these results also raised the need for further studies focusing on the pharmacologic treatment aimed at increasing myocardial microcirculation function, which can possibly improve myocardial function in CTD patients.

GLPS has been proven to have crucial clinical value with a high reproducibility [40, 41]. Claus et al. [40] have reported that GLPS can be used to identify the patients with subclinical myocardial dysfunction with a particular value. The data acquired from the ROC analysis identified that GLPS was the best indicator for differentiating CTD patients from normal controls, which was analogous with the report of Claus et al.

Several limitations exist in our research. Firstly, this was a single-center and retrospective study, and the likelihood for selection bias cannot be disregarded. Secondly, we used techniques focusing on the combination of tissue tracking, first-pass perfusion, and LGE. Future mapping techniques such as T1 mapping and T2 mapping are expected to be used in the further research in evaluating the cardiac involvement in CTD patients. Finally, follow-up data of CTD patients were not included. Further follow-up studies will be performed to understand the evolution of myocardial injury so as to provide reliable imaging evidence for early clinical diagnosis and treatment in patients with CTD.

## Conclusions

In conclusion, CTD patients showed impaired LV global myocardial deformation, microcirculation perfusion, and presence of LGE. Cardiac involvement might be more severe in non-IIM patients than in IIM patients. The impaired microvascular perfusion and the presence of LGE were associated with LV deformation in CTD patients. Early screening of CTD patients with cardiac involvement using CMR would be significant for timely treatment.

### Supplementary Information

Below is the link to the electronic supplementary material.

Supplementary file1 (DOCX 19 kb)

## Data Availability

The datasets used and analyzed during the current study are available from the corresponding author on reasonable request.
