# Supplementary material for: Quantitative assessment of left ventricular myocardial involvement in patients with connective tissue disease: a 3.0T contrast-enhanced cardiovascular magnetic resonance study
Source: Int J Cardiovasc Imaging. 2022 Mar 13;38(7):1545–54. doi: 10.1007/s10554-022-02539-6 (PMC11143006; doi:10.1007/s10554-022-02539-6)
Supplement: Supplementary file 1 — Supplementary file1 (DOCX 19 kb) [file 10554_2022_2539_MOESM1_ESM.docx]

**Supplementary Material:**

**The detailed diagnostic criteria for CTD were as follows:**

IIM: Diagnosis of IIM was based on the criteria of the Mediator of the European Neuromuscular Centre (ENMC) workshop [17].

SLE: Diagnosis of SLE was based on the criteria of the 1982 American College of Rheumatology [18].

RA: Diagnosis of RA was based on the criteria of the 1987 American College of Rheumatology (modified in 2010) [19].

SSc: Diagnosis of SSc was based on the criteria of the 2013 American College of Rheumatology/ European League against Rheumatism [20].

Sjogren's syndrome：Diagnosis of Sjogren's syndrome was based on the revised criteria of the 2002 American European Consensus Group [21].

Mixed connective tissue disease: Diagnosis of mixed connective tissue disease was based on the modified Sharp’s criteria [22].

Undifferentiated connective tissue disease: Diagnosis of undifferentiated connective tissue disease was based on the following criteria [23]: 1) symptoms and signs suggesting of an autoimmune disease but failing to meet criteria for a definite CTD; 2) patients having at least one positive autoantibody.
